# Enhancing feedback by health coaching: the effectiveness of mixed methods approach to long-term physical activity changes in nurses. An intervention study

**DOI:** 10.1186/s12912-024-01815-1

**Published:** 2024-03-22

**Authors:** Agnieszka Nerek, Katarzyna Wesołowska-Górniak, Bożena Czarkowska-Pączek

**Affiliations:** Department of Clinical Nursing, Medical University of Warsaw, Ciołka 27, 01-445 Warsaw, Poland

**Keywords:** Daily number of steps, Health Coaching, Health-promoting Behaviours, Long-term changes, Nursing staff, Physical activity

## Abstract

**Background:**

Although knowledge of the barriers and motivators to physical activity participation among nurses is increasing, the factors influencing motivation methods’ effectiveness are not completely defined. This study aimed to identify the methods that support increasing the level of daily physical activity and the factors that influence the effectiveness of motivation methods among nurses.

**Methods:**

This study was based on an intervention study protocol. All registered nurses in clinical settings were invited to participate in the study. The study involved 71 professionally active nurses. A self-reported questionnaire was used to collect sociodemographic and employment data. The level of physical activity was assessed using the International Physical Activity Questionnaire, and the daily number of steps was assessed using a pedometer. Body composition was measured using a bioimpedance method, and the 5-year risk of cardiovascular events was assessed using the Harvard Score. The intervention included self-monitoring daily steps using a pedometer and completing a diary daily for one month. Additionally, a few-minute speech was sent to each participant via email on the intervention’s 7th, 14th, and 21st days.

**Results:**

The analysis revealed a higher value of physical activity recorded in the follow-up compared to the initial and final measurement in the Recreation domain [Met] (*p* < 0.001) and a higher value of daily steps in the follow-up compared to the final measurement (*p* = 0.005). Participants with a higher Harvard Score were more likely to increase their daily number of steps (OR = 6.025; 95% CI = 1.70-21.41), and nurses working in hospital wards were less likely to do so (OR = 0.002; 95% CI = 0.00-0.41).

**Conclusions:**

Recommendations for physical activity in the nursing population should focus on increasing leisure time physical activity and regular risk assessment of cardiovascular events. A mixed methods approach, such as feedback enhanced by health coaching, effectively achieves long-term physical activity changes in nurses.

**Supplementary Information:**

The online version contains supplementary material available at 10.1186/s12912-024-01815-1.

## Background

The benefits of regular physical activity are well-documented and include improved cardiovascular function and musculoskeletal strength, reduced morbidity and mortality risk due to chronic disease, and decreased risk of mental health problems [1, 2]. Additionally, performing physical activity can reduce work-related stress and incidence of burnout [3–5] and positively affect emotional intelligence and resilience [6, 7]. Moreover, the health-related behaviors of medical professionals are associated with their quality of life [8].These benefits are increasingly emphasized in research on healthcare worker populations [9]. Despite being aware of the benefits, many nurses have low levels of physical activity [10–13], placing them at an increased risk for chronic diseases [14] andleading to increased absences and decreased work capacity, potentially increasing the workload for other nurses on the unit [15, 16]. Furthermore over 30% of registered nurses are overweight or obese [9, 17, 18], which may result from stressful work conditions. Such a relationship has previously been found among nurses [19]. Moreover, rotating night shift work is related with unhealthy lifestyles and both are involved in increasing the risk of type 2 diabetes. Rotating night shift work alters sleep and circadian rhythms that play important roles in daily normal metabolic function. Disruption of sleep and circadian rhythms are involved in lifestyle behaviors such as smoking, diet, physical activity, they also could disrupt the intestinal microbiota, which has the role in development of metabolic diseases. Some researchers suggest that most cases of type 2 diabetes could be prevented by adherence to a healthy lifestyle, and the benefits would be larger in rotating night shift workers [20]. Although nurses have the highest rates of obesity and overweight, they have the lowest participation in workplace health promotion activities among all healthcare professional groups working in hospitals [21]. As such, nurses should be a target group for health promotion initiatives [22].

Several methods have been identified to promote physical activity andthe workplace is an ideal setting to implement health promotion initiatives to reduce noncommunicable disease risk factors, according to the World Health Organization [23]. On the other hand, theresults of interventions regarding physical activity promotion in nurses, especially workplace initiatives, are inconsistent [14]. The quality of studies assessing the impact of such interventions is mostly low to moderate, and results should be interpreted cautiously [14]. Moreover assessing the physical activity using one method– only subjective or objective one may have discrepancy. Self-reported measures tend to overestimate physical activity levels when compared with objective assessments [22, 24]. Effective methods in increasing nurses’ physical activity include self-monitoring using the accelerometer or physical activity challenges, but the rate of change decreases over time [5, 25]. Visual triggers and health coaching with texting have also increased physical activity levels [26]. Based on replicable behavior change techniques, self-monitoring behavior and subsequent feedback are typically an effective combination of methods to improve nurses’ physical activity [27]. One recommendation to increase the level of physical activity is to remove barriers that discourage or prevent nurses from engaging in physical activity. These barriers include lack of time, excessive work, irregular shifts, stress, exhaustion, and fear of pain after exercise, which results from the physically demanding nature of the nursing profession [28–30]. The nursing profession has long been considered physically demanding [31] and is associated with a very high prevalence of musculoskeletal disorders [32]. Previous studies have confirmed that one of the biggest barrier to motivating nurses to increase their physical activity is the fear of pain that may occur after exercise [22]. In the nursing population, high fear-avoidance beliefs regarding physical activity have been significantly associated with experiencing chronic, disabling low back pain [33].

Many authors report the necessity to investigate personal and occupational factors that could help nurses sustain physical activity levels in the long term [22, 25]. This study aims to identify the methods that support increasing the level of daily physical activity and the sociodemographic, occupational, and health-related factors that influence the effectiveness of motivation methods in increasing daily physical activity among nurses.

## Methods

### Design and settings

This study was based on an intervention study protocol, and data were collected over 10 months, from September 2021 to June 2022. The inclusion process was continuous and intentionally included different seasons to account for the variability of the daily number of steps depending on the season, which is confirmed in the literature [34]. All registered nurses in clinical settings were invited to participate in the study. Detailed information about the study was disseminated in hospitals and outpatient clinics in Warsaw, and a full list of participating institutions is included in Appendix 1. The Strengthening the Reporting of Observational Studies in Epidemiology (STROBE) Statement was used to report data [35], along with the Page et al. statement about improving the reporting of therapeutic exercise interventions in rehabilitation research [36].

### Sample

The inclusion criteria for this study were being a professionally active nurse working in a clinical setting, being able to walk unassisted, being willing to wear a monitoring device on the wrist, and having access to the Internet and an email address. Criteria for exclusion from participation in the study were dysfunction or disability affecting gait locomotion, pregnancy, medical contraindications to exercise, or implanted pacemakers or other devices contraindicated for body composition assessment using the bioimpedance method. Sample size analysis was performed using G*Power 3.1.9.4 software. Based on analysis of variance (ANOVA) results for a moderate effect size (f = 0.25), alpha = 0.05, and test power at 0.95, the sample size required was 43 participants for repeated measures.

### Data sources and measurements

The study was divided into three phases: inclusion, intervention with final assessment, and follow-up measurements. During the inclusion process, all participants consented to participate in the study. A self-reported questionnaire was used to collect sociodemographic data such as sex, age, and place of residence, as well as professional activity-related data such as education, clinical specialization, management position, number and type of workplace(s), total monthly workload, type of shift, and work experience.

Body composition, including Body Mass Index (BMI) [kg/m^2^], the absolute value of Fat Mass (Fat) in kg, and the absolute value of Free Fat Mass (FFM) in kg, was measured using a bioimpedance method (Body Composition Analyzer Maltron Bioscan 920, UK). The measurement was taken during rest in the supine position after measuring the participant’s body weight in kg. Blood pressure was measured once in the supine position using an upper arm automatic blood pressure monitor (Omron M4). On average, each examination lasted up to 10 min for each person.

The 5-year risk of cardiovascular events was assessed using the Harvard Score (Score), a non-laboratory method shown to predict cardiovascular events as accurately as the Framingham Coronary Heart Disease Risk Score, which requires laboratory-based values. The Harvard Score utilized non-laboratory-based risk factors such as age, sex, diabetes status (no diabetes or diabetes), current smoking status (non-smoker or smoker), systolic blood pressure, and Body Mass Index to determine 5-year cardiovascular disease risk categories: <5% (low), 5–10% (low), > 10–20% (moderate), > 20–30% (high), or > 30% (high). Cardiovascular risk scores were not calculated for participants younger than 35 [37].

The level of daily physical activity was assessed using a Polish version of the long form of the International Physical Activity Questionnaire (IPAQ). The questionnaire was structured to provide separate domain-specific scores for walking (total walking MET), moderate-intensity activity (total moderate MET), and vigorous-intensity activity (total vigorous MET) within each of the work (Occupational activity [MET]), active transportation (Active locomotion [MET]), domestic chores (Domestic chores [MET]), and leisure-time domains (Recreation [MET]). Total time engaged in walking, moderate physical activity, and vigorous physical activity, as well as the total level of weekly activity (total physical activity score - TPAS), were computed according to the guidelines [38].

**Fig. 1:**
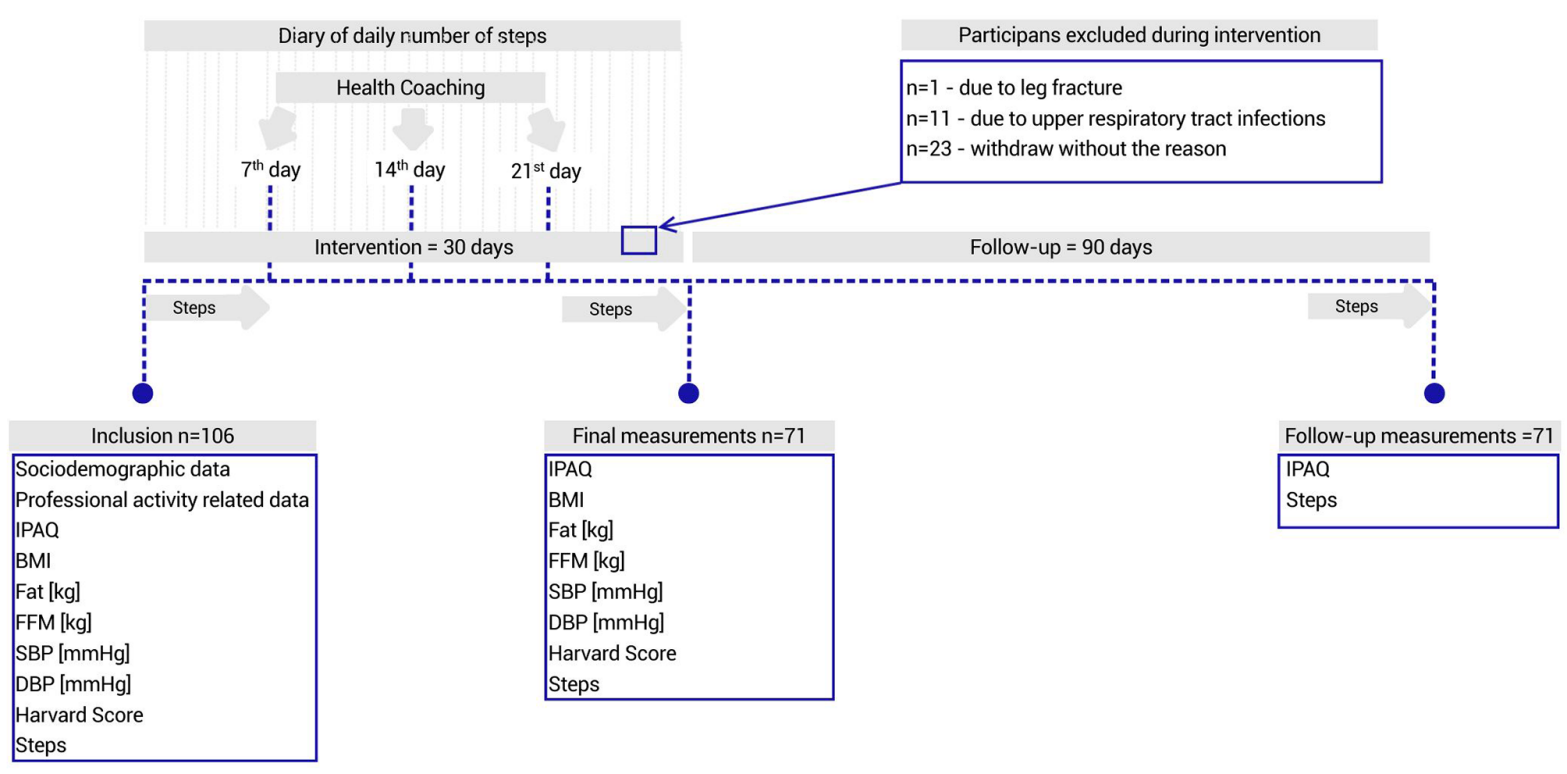
A flow chart depicting the measures taken in each study phase

The average daily number of steps was measured using a pedometer Health Manager App Beurer AS 80 (model 2016/2017) for 7 consecutive days before the intervention phase. The nurses were instructed to wear the activity monitor from waking to bedtime (except during water activities) and to complete a diary of their daily number of steps. The pedometers had a memory function and researchers cross-checked the data stored in the pedometer’s memory with the participant’s diaries. A flow chart depicting the measures taken in each study phase is presented in Fig. 1.

### Intervention

All participants wore the pedometer for one month and had to complete a diary of their daily number of steps. In addition, on the intervention’s 7th, 14th, and 21st days, motivation for physical activity participation was enhanced through a few-minute speech recorded and sent to each participant’s email. The researchers have checked if each participant has read the email. The speeches covered topics such as guidelines for daily physical activity, the impact of physical activity on health, the health effects of physical inactivity, and tips on gradually increasing daily physical activity. During the speeches, participants were encouraged to achieve the goal of 10,000 steps per day, but goal achievement was not the purpose of the study. At the end of the intervention phase, final measurements were conducted, including the level of daily physical activity measure, the average daily number of steps, body composition, and Harvard Score.

### Follow-up

The follow-up assessments were conducted after 3 months of final measurements and included the level of daily physical activity measure and the average daily number of steps.

### Statistical methods

All data were analyzed using IBM SPSS Statistics, version 28.0. Descriptive statistics were used to assess sample characteristics. The Shapiro-Wilk test was used to assess the consistency of the quantitative variable with a normal distribution. For the comparison of two related samples and quantitative variables, the t-test was performed, and for more than two measurements, the Friedman test or the analysis of variance for repeated measures was used (e.g., comparison of the level of daily physical activity measure and Steps values between each study phase). To determine which of the analyzed variables were predictors of the decrease/increase in the average daily number of steps (Steps), a logistic regression analysis was performed using the backward elimination method with maximum likelihood estimation. The model explained a total of 21.9% of the variance of the dependent variable (Cox and Snell R2 = 0.219) and was well fitted to the data, χ2 [8] = 3.90; *p* = 0.866 (Hosmer-Lemeshow test). Spearman’s rho correlation analysis determined the relationships between quantitative variables. The significance level was set at α = 0.05.

## Results

### Participant characteristic

A total of 106 professionally active Polish nurses were included in the study, and 71 completed all stages. One participant was excluded during the intervention due to a leg fracture, 11 were excluded due to upper respiratory tract infections, and 23 withdrew without reason. The detailed characteristics of the study participants are summarized in Table 1. Participants were predominantly middle-aged (mean 35.65 ± 10.40 [years]), female (85.9%), residing in a city (91.5%), and had a master’s degree (59.2%) without clinical specialization (69%). Most of the participants were employed in hospital settings (90.1%), working overtime (62%) on mixed shifts (81.7%). The average work experience of participants was 12.3 ± 9.28 [years].

**Table 1:** The detailed characteristics of the study participants

Variables:	M (± SD)/ n(%)
*Sociodemographic Variables*:	
Sex, n (%)	
Woman	61 (85.9)
Man	10 (14.1)
Age [years], M (± SD)	35.65 (± 10.40)
Place of residence, n (%)	
City	65 (91.5)
Village to medium-sized town	6 (8.5)
Professional activity related variables:	
Education, n (%)	
Bachelor of Nursing	29 (40.8)
Master of Nursing	42 (59.2)
Clinical Specialization, n (%)	
Yes	22 (31)
No	49 (69)
Management position, n (%)	
Yes	5 (7)
No	66 (93)
Workplace, n (%)	
Hospital Ward	64 (90.1)
Other	7(9.9)
Total monthly workload, n (%)	
Full-time work	27 (38)
More than full-time (full-time and overtime)	44 (62)
Shift type, *n* (%)	
Daily shift	13 (18.3)
Rotating shift	58 (81.7)
Work experience [years], M (± SD)	12.3(± 9.28)

M: Mean; ± SD: Standard Deviation; Village to medium-sized town: <100,000 inhabitants; City: >100,000 inhabitants; Daily shift: working during the day; Rotating shift: working both day and night shifts

### Physical activity before and after intervention

There were no significant differences in the Total Physical Activity Score (TPAS [MET]) assessed by IPAQ between study points, but a detailed analysis revealed significant differences in the Recreation domain [MET]. The follow-up recorded a higher value than the initial and final measurements. However, the differences between the initial and final measurements were not significant. The analysis showed significant differences between the measurements for ‘Domestic chores’ but post hoc analysis using the Dunn test with Bonferroni correction of the significance level did not show significant differences between the groups.

**Fig. 2:**
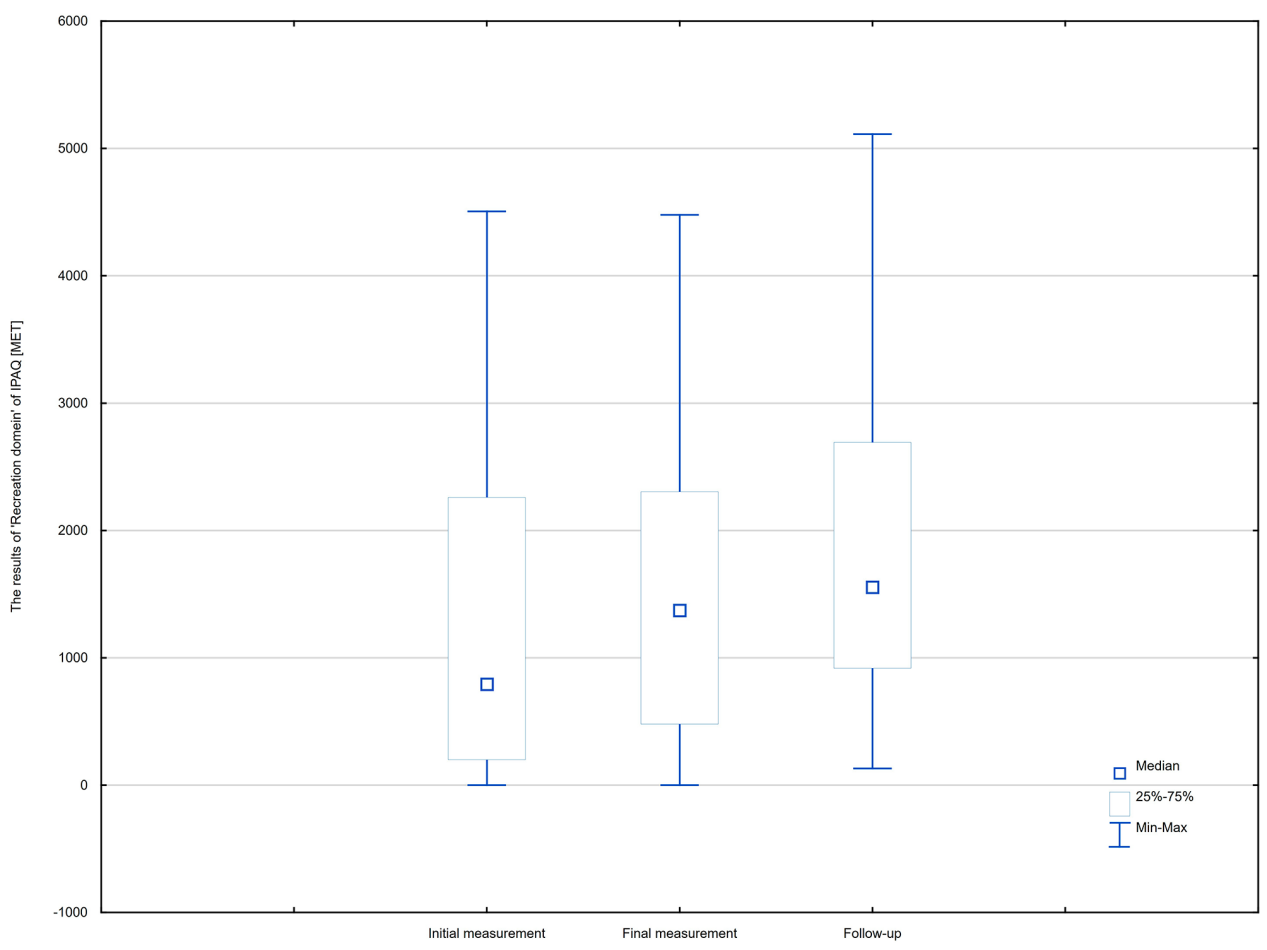
The results of Recreation domain [MET] in all phases of the study

**Fig. 3:**
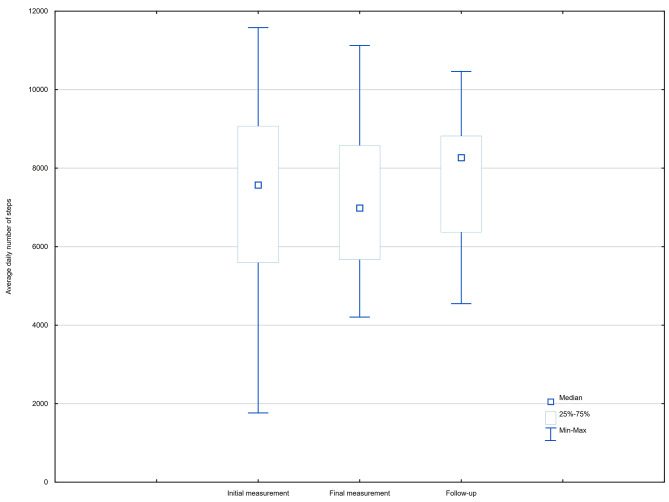
The results of the average daily number of steps [Steps] in all phases of the study

The average daily number of steps (Steps) was significantly higher in the follow-up compared to the final measurement, but there were no differences between the initial and final measurements. The comparisons of the results of IPAQ and Steps between each study phase are presented in Table 2 and in Figs. 2 and 3.

**Table 2:** Comparison the results of IPAQ and Steps between each study phase

	Initial measurement (*n* = 71)			Final measurement (*n* = 71)			Follow-up (*n* = 71)			χ2/F	*p*
	M	Me	SD	M	Me	SD	M	Me	SD		
IPAQ: Total Physical Activity Score [MET]	30916.00	25104.00	20762.50	26395.36	20704.00	14222.73	25840.25	23970.00	9053.79	0.20	0.906
IPAQ: Occupational activity [MET]	19102.94	14652.00	14214.43	15943.76	12264.00	10346.49	16099.00	15015.00	6166.47	1.38	0.502
IPAQ: Active locomotion [MET]	4926.06	2772.00	4989.03	3637.19	2916.00	2638.64	3838.65	3465.00	2605.67	3.19	0.202
IPAQ: Domestic chores [MET]	4640.60	3480.00	4649.33	4640.35	4020.00	3532.43	3647.25	3360.00	2051.15	6.33	0.042
IPAQ: Recreation [MET]	2246.40	792.00	3514.89	2165.99	1371.00	3046.46	2240.23	1554.00	2196.21	17.51	< 0.001*
Steps	7267.68	7571.00	2066.31	7107.04	6985.00	1763.73	7764.73	8268.00	1571.87	5.43	0.005**

*Significant difference between ‘Follow-up’ and ‘Initial measurement’ and significant defference between ‘Follow-up’ and ‘Final measurement’; ** Significant difference between ‘Follow-up’ and ‘Final measurement’

M: Mean; Me: Median; ± SD: Standard Deviation; IPAQ: International Physical Activity Questionnaire; Steps: An average daily number of steps; *p*: *p*-value

**Fig. 4:**
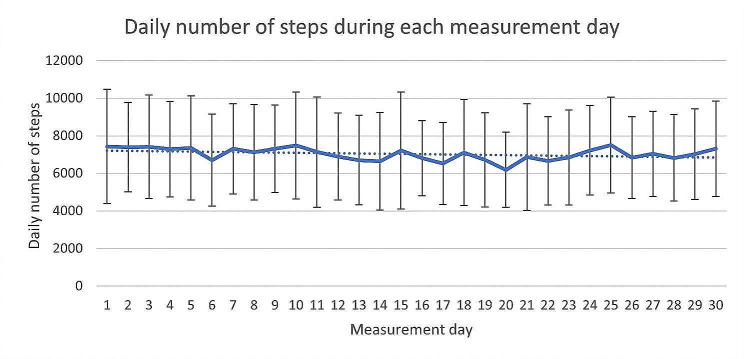
The trend of the daily number of steps during the intervention phase

The trend analysis of the Step number revealed that the average daily number of steps was similar during each of the 30 days of the intervention. A slight decrease from the average was observed on the 6th, 14th, 17th, and 20th day of the study, while a higher activity level was observed on the 25th day of the study. Figure 4 depicts the trend of the daily number of steps. In this figure, the whiskers represent the standard deviation, the solid line indicates the average number of steps for each measurement day, and the dashed line represents the average daily number of steps over the entire analyzed period.

### Body composition and cardiovascular disease risk before and after intervention

The study revealed a significant decrease in Body Mass Index and the absolute value of Fat Mass at [kg] in the final measurement compared to the initial measurement (t = 2.09; *p* = 0.04 and t = 2.22; *p* = 0.03, respectively). The comparisons of the results of blood pressure, body composition, and Harvard Score between each study phase are summarized in Table 3.

**Table 3:** Comparisons the results of blood pressure, body composition and Harvard Score between each study phase

	Initial measurement (*n* = 71)		Final measurement (*n* = 71)		*t*	*p*	95% CI		Cohens d
	M	SD	M	SD			LL	UL	
DBP [mmHg]	75.13	7.81	75.59	7.32	-1.57	0.121	-1.06	0.13	0.19
SBP [mmHg]	119.59	9.03	119.94	8.10	-1.09	0.281	-1.00	0.29	0.13
BMI	24.62	4.70	24.60	4.71	2.09	**0.040**	0.00	0.04	0.25
Fat [kg]	19.80	8.88	19.63	8.93	2.22	**0.030**	0.02	0.33	0.26
FFM [kg]	49.38	8.88	50.15	9.24	-1.96	0.054	-1.55	0.01	0.23

M: Mean; ± SD: Standard Deviation; *t*: *t*-test; *p*: *p*-value; CI: Confidence interval; DBP: Diastolic Blood Pressure; SBP: Systolic Blood Pressure; BMI: Body Mass Index; Fat[kg]: absolute value of Fat mass; FFM [kg]: absolute value of Free Fat Mass

Factors influencing the increase in the average daily number of steps after the intervention were examined in the present study. The results showed that 43.7% of participants had increased the Steps number at the final measure and 63.4% at the follow-up. Logistic regression analysis revealed that the odds for an increase in the Steps number decreased with higher systolic blood pressure (SBP) [mmHg] (OR = 0.92; 95% CI = 0.85-1.00) and with working in a hospital ward (OR = 0.002; 95% CI = 0.00-0.41). On the other hand, the odds for an increase in the Steps number increased with a higher Harvard Score (OR = 6.025; 95% CI = 1.70-21.41) and Free Fat Mass (FFM) [kg] (OR = 1.451; 95% CI = 1.07–1.96) measures. None of the other sociodemographic orvocational variables, were significant predictors of an increase or decrease in the participants’ number of steps. The model explained a total of 21.9% of the variance in the explained variable (Cox and Snell R2 = 0.219) and was a good fitted to the data, χ2 [8] = 3.90; *p* = 0.866 (Hosmer-Lemeshow test). The detailed results of analysis are presented in Table 4.

**Table 4:** Logistic regression coefficients for the model explaining the decline/increase of Steps

Variable	B	SE	Wald	p	OR	95% CI for OR	
						LL	UL
Working place [Hospital ward]	-6,03	2,63	5,27	0,022	0,002	0,00	0,41
SBP [mmHg]	-0,08	0,04	4,13	0,042	0,920	0,85	1,00
Score	1,80	0,65	7,71	0,006	6,025	1,70	21,41
TBW [l]	-0,45	0,20	5,00	0,025	0,641	0,43	0,95
Fat %	-3,27	1,41	5,41	0,020	0,038	0,00	0,60
FFM [kg]	0,37	0,16	5,79	0,016	1,451	1,07	1,96
FFM %	-3,30	1,43	5,36	0,021	0,037	0,00	0,60

B: estimated coefficient; SE: standard error; *p*: *p*-value; OR: odds ratio; CI: Confidence interval; SBP: Systolic Blood Pressure; Score: Harvard Score; TBW: Total Body Water; Fat%: FFM: Free Fat Mass; FFM%: Body Fat Percentage; FFM%: Free Fat Mass Percentage

## Discussion

A mixed method approach, using a pedometer and 3 motivational speeches did not influence the Total Physical Activity Score (TPAS [MET]) assessed by IPAQ, but a detailed analysis revealed a significant increase in the Recreation domain [MET], where a significantly higher value was recorded in the follow-up compared to the initial and final measurements. Furthermore, the average daily number of steps was significantly higher in the follow-up compared to the final measurement, suggesting that the intervention had a long-lasting effect that persisted after the completion of the intervention phase. This finding is inconsistent with previous studies that reported short-lived increases in physical activity after web-based interventions providing feedback and physical activity challenges [39]. The results suggest that other motivation methods, such as health coaching, should enhance interventions with feedback and physical activity challenges [26]. The good match of health coaching applied in the present study, which occurred just after the decrease in the daily number of steps, supports this hypothesis. Drawing on evidence-based behavior change techniques, such as coaching, social support, feedback, barrier identification, follow-up prompts, and health checks may help reinforce long-term physical activity changes [40].

Our findings suggest that the Recreation domain of daily physical activity is the most susceptible to change. This aligns with our previous research, which demonstrated that nurses who are more motivated to be active engagement in a higher level of leisure-time physical activity than those who are less motivated [22]. Research has also shown that engaging in moderate- to vigorous-intensity physical activity before a morning shift is associated with increased sedentary time and decreased physical activity during work hours [41]. Henwood et al. found that nurses who engaged in ≥ 30 min/day of moderate workplace activity were not healthier than those who found the same amount of physical activity during their leisure time. They concluded that workplace activity does not positively affect health and well-being [42]. Parker et al. suggested that occupational physical activity may not provide the same health benefits as leisure-time physical activity for nurses [43]. Furthermore, Richard et al. reported that leisure-time physical activity is inversely associated with all-cause mortality, whereas occupational physical activity does not have clear associations [44]. These observations support the effectiveness of intervention programs that promote physical activity, particularly in the leisure-time domain, which is most recommended for health benefits. Providing sufficient time for recovery after work and ensuring compliance with ergonomic principles are crucial to enable nurses to engage in leisure-time physical activity. Our study also revealed a significant change in participants’ Body Mass Index, which may indicate that the motivational strategies employed during the intervention phase influenced other healthy behaviors besides physical activity. In our study strength of the effect is low, but other authors have also confirmed the effectiveness of health coaching in promoting behavior changes for improved health, including body weight loss, increased physical activity, mental health status, enhanced medication adherence, better social support, and improved physical health status, including HbA1c. Health coaching is also a low-cost tool [45].

The presented study identified several factors predisposing individuals to increase their daily number of steps. Participants with higher Harvard Scores were likelier to increase their Steps number, which contradicts the belief that fear arousal induced by threat (future punishment) is likely counterproductive when self-efficacy is low [27]. This suggests that nurses with higher knowledge about the consequences of chronic diseases may be more motivated to change their health behaviors because of the fear of threat (future punishment), such as the 5-year risk of cardiovascular events. Conversely, participants with higher Free Fat Mass [kg] are more vulnerable to applied motivation, suggesting that naturally active individuals in good physical condition are more willing to engage in physical activity. Nurses who agree that physical activity positively affects their mental and physical condition were more motivated to engage in physical activity and showed a higher level of leisure-time physical activity [22]. However, working in a hospital ward harmed the increase in the number of steps after the applied intervention. Although the type of hospital ward was not distinguished in the study, other research has shown that the average number of steps and distance traveled was greatest for nurses working in the emergency room, followed by the intensive care unit, surgical ward, and medical ward [46]. Working in a hospital ward and engaging in direct patient care is considered the most demanding [41, 47], and nurses not involved in direct patient care are more sedentary [47]. Nurses working in rotating shifts showed a significantly higher level of general physical activity than nurses working only in daily shifts [22, 48]. Furthermore, nurses who are highly active during work hours are less likely to engage in leisure-time physical activity, as confirmed by Chappel et al., who revealed that occupational walking time was associated with lower activity levels during leisure time [41]. Although working in a hospital ward is difficult to modify, ensuring appropriate time for recovery and compliance with ergonomic principles is necessary to enable nurses to increase their leisure-time physical activity.

### Limitations

One limitation of the presented study is the possibility that participants may have adopted a healthier lifestyle during the observation period than they normally would have, knowing that their physical activity was being recorded. Therefore, an observer effect cannot be ruled out, a common limitation in similar studies [49]. For the same reasons, a control group was not included in the study. Another limitation is self-selection, meaning nurses not interested in increasing their physical activity may have chosen not to participate in the study. Moreover, the adherence to listening to a few-minute speeches was not assessed by a reliable method and it was based solely on participant’s self-reporting. Researchers only confirmed whether each participant had read the email.

### Strengths

One strength of the study was the utilization of both objective and subjective methods to assess daily physical activity. The study utilized the IPAQ questionnaire, allowing for a comprehensive analysis of physical activity. This analysis included not only fundamental calculations derived from the assessment but also additional domains such as recreation, household duties, and duties related to professional work. The sample size was well-matched to the intervention. Moreover, the recommended bio-psycho-social model was used in the logistic regression analysis, taking into account metric, professional, social and health-related factors influencing the increase/decrease of daly physical activity.

## Conclusions

To reinforce long-term changes in nurses’ physical activity, employing mixed methods, such as using a pedometer and delivering motivational speeches, seems a promising way to proceed. Feedback and physical activity challenges should be supplemented by other motivational techniques, such as health coaching, effectively promoting behavior changes for improved health. It should be highly recommended because leisure-time physical activity is the most susceptible to change according to motivational techniques. Participants with higher Harvard Scores were more likely to increase their daily number of steps; therefore, regular evaluation of this indicator among nurses is warranted. Working in a hospital ward had the most negative impact on increasing the daily number of steps, and this factor is difficult to modify. Therefore, ensuring appropriate time for recovery and compliance with ergonomic principles is necessary to enable nurses to increase their leisure-time physical activity.

### Electronic supplementary material

Below is the link to the electronic supplementary material.

Supplementary Material 1

## Data Availability

The datasets used and/or analysed during the current study are available from. the corresponding author on reasonable request.
