# Supplementary material for: Enhancing feedback by health coaching: the effectiveness of mixed methods approach to long-term physical activity changes in nurses. An intervention study
Source: BMC Nurs. 2024 Mar 22;23:196. doi: 10.1186/s12912-024-01815-1 (PMC10958873; doi:10.1186/s12912-024-01815-1)
Supplement: Supplementary file 1 — Supplementary Material 1 [file 12912_2024_1815_MOESM1_ESM.docx]

Appendix 1. Full list of institutions that participated in the study.

1. University Clinical Centre of the Medical University of Warsaw Children’s Hospital (Uniwersyteckie Centrum Kliniczne Warszawskiego Uniwersytetu Medycznego Dziecięcy Szpital Kliniczny im. Polikarpa Brudzińskiego) Warsaw
2. Medicover Multidisciplinary Hospital (Wielospecjalistyczny Szpital Medicover), Warsaw
3. Medical Centre (Centrum Medyczne), Warsaw
4. University Clinical Centre of the Medical University of Warsaw Central Teaching Clinical Hospital (Uniwersyteckie Centrum Kliniczne Warszawskiego Uniwersytetu Medycznego Centralny Szpital Kliniczny), Warsaw
5. Warsaw Medical Care Center ‘KOPERNIK’ (Warszawskie Centrum Opieki Medycznej "KOPERNIK"), Warsaw
